# Respiratory system elastance monitoring during PEEP titration

**DOI:** 10.1186/cc10710

**Published:** 2012-03-20

**Authors:** YS Chiew, JG Chase, GM Shaw, T Desaive

**Affiliations:** 1 University of Canterbury, Christchurch, New Zealand; 2 Christchurch Hospital, Christchurch, New Zealand; 3 University of Liege, Belgium

## Introduction

PEEP selection during mechanical ventilation (MV) for patients with acute lung injury (ALI) and acute respiratory distress syndrome (ARDS) remains a challenge for clinicians. Clinicians often rely on experience and intuition in setting MV, resulting in a more variable treatment and outcome. We hypothesise that monitoring patient-specific respiratory system elastance (Ers) during PEEP change may provide an insight into the patient's condition.

## Methods

Thirteen patients with ALI/ARDS underwent a step-wise PEEP increase (5 cmH_2_O) recruitment manoeuvre (RM) until peak airway pressure reaches 45 cmH_2_O. Airway pressure and flow profile were recorded using a pneumotachometer. The change of patient's respiratory system elastance (Ers = 1/compliance) and the end of expiratory lung volume (EELV) during RM were estimated and studied. The trials were approved by New Zealand South Island Regional Ethics Committee.

## Results

**Figure 1:**
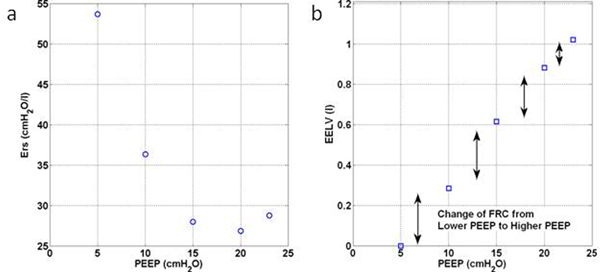
**Ers and EELV change with PEEP increase**.

The median (IQR) Ers over all patients was 34.0 cmH_2_O/l (26.1 to 51.0), reflecting the heterogeneity of the patients and their response to PEEP. This outcome supports the idea that MV/PEEP should be individualised. During RM, patients' Ers decreased with PEEP increase until a specific minimum and increase at higher PEEP. The decreased of Ers suggest alveolar recruitment whereas an increase of Ers at higher PEEP shows potential overinflation. An example is shown in Figure 1a. A clear inflection/minimum Ers can be found in Figure 1a, indicating a potential method to optimise PEEP selection for a particular patient. Figure 1b shows the change of patient's EELV with PEEP increase. As PEEP increases, the potentially recruitable collapsed lung decreases.

## Conclusion

The change of patient-specific Ers and EELV during minimally invasive PEEP titration provides an insight into the patient's lung condition, and thus could potentially be used as a method to individualise MV treatment and, in particular, PEEP selection.

